# Lipid and glucose alterations in perinatally-acquired HIV-infected adolescents and young adults

**DOI:** 10.1186/s12879-015-0853-8

**Published:** 2015-03-08

**Authors:** Daniel Blázquez, José Tomás Ramos-Amador, Talía Saínz, María José Mellado, Marta García-Ascaso, María Isabel De José, Pablo Rojo, María Luisa Navarro, María Ángeles Muñoz-Fernández, Jesús Saavedra, Miguel Angel Roa, Santiago Jiménez, José Beceiro, Luis Prieto, Milagros García Hortelano, María Isabel González-Tomé

**Affiliations:** Pediatric Infectious Diseases Unit, 12 de Octubre University Hospital Complutense University, Instituto de Investigación Hospital 12 de Octubre, C/Avenida de Córdoba, S/N 28048 Madrid, Spain; Department of Pediatrics, Clínico San Carlos Hospital, Madrid, Spain; Unidad de Pediatría Hospitalaria E. Infecciosas y Tropicales Pediátricas, La Paz University Hospital, Madrid, Spain; Department of Pediatrics, Puerta de Hierro University Hospital, Madrid, Spain; Pediatric Infectious Diseases Unit, Gregorio Marañón University Hospital, Madrid, Spain; Laboratorio de Inmunobiología Molecular, Instituto de Investigación Sanitaria Hospital Gregorio Marañón (IISHGM), Madrid, Spain; Department of Pediatrics, Móstoles University Hospital, Móstoles, Spain; Department of Pediatrics, Principe de Asturias University Hospital, Alcalá de Henares, Spain; Department of Pediatrics, Getafe University Hospital, Getafe, Spain

**Keywords:** HIV, Adolescents, Insulin resistance, Waist, Lipid abnormalities

## Abstract

**Background:**

Successful antiretroviral therapy (ART) has dramatically reduced mortality among HIV-infected children. However, there is growing concern about long-term effects associated to ART. The aim of this study was to determine the prevalence of metabolic abnormalities in a cohort of perinatally HIV-infected adolescents and young adults and to identify associated factors.

**Methods:**

We present results from a cross-sectional analysis including individuals 12 to 20 years of age, from a prospective, longitudinal cohort of perinatally-acquired HIV-infected children, adolescents and young adults in Madrid. Clinical and immunological data were recorded and complete lipid and glycemic profiles were determined.

**Results:**

Ninety-nine adolescents were included, with a median age of 15.3 years [13.6-16.7]. Patients with abnormal levels of lipids were as follows: 27.2% total cholesterol ≥200 mg/dl, 25.9% LDL cholesterol (LDL-c) ≥ 130 mg/dl, 14.1% HDL-C < 35 mg/dl and 39.8% triglycerides ≥ 150 mg/dl. Current use of protease inhibitors (PI) was associated with higher triglyceride values (p = 0.022). Four (4.6%) patients showed fasting glucose ≥ 100 mg/dl and 30.6% presented with insulin resistance (IR) (HOMA-IR over the 90th centile). In the multivariate logistic regression analysis adjusted for sex, age, weight, Tanner stage, protease inhibitors (PI) and nucleoside reverse transcriptase inhibitors (NRTI) treatment length and CD4 nadir, IR was associated with higher waist circumference Z score; OR: 3.92(CI95%: 1.15-13.4) (p = 0.03).

**Conclusion:**

There was a high prevalence of insulin resistance and lipid abnormalities in this cohort of perinatally-acquired HIV-infected adolescents. A simple clinical measurement like waist circumference Z score might be a reliable marker and predictor of insulin resistance in these patients.

## Background

Life expectancy has dramatically improved in HIV-infected children since the introduction of highly active antiretroviral therapy (HAART). However, several studies have reported a high prevalence of metabolic disorders in this unique population, and there is growing concern about the long-term complications of lifelong exposure to ART. Dyslipidemia and insulin resistance (IR) have been widely described among ART-treated children and adolescents, although the prevalence of these disorders differs significantly among studies [1-4].

In the general population, metabolic disorders during childhood, especially dyslipidemia and IR, have been associated with early development of cardiovascular disease (CVD) [5]. HIV-infected subjects are known to be a population at high risk for premature atherosclerosis [6,7] and in fact, previous studies have already demonstrated the presence of subclinical atherosclerosis in HIV-infected children and adolescents [8-10]. Although the underlying mechanisms are not well understood, ART-mediated dyslipidemia has been proposed as a determinant factor [11]. It has been shown that HIV can by itself decrease the insulin sensitivity of peripheral tissues [12] and a close association between glucose tolerance and coronary atherosclerosis has been well established [13]. Despite the growing concern regarding the increased risk of metabolic complications and early cardiovascular disease associated to HIV infection, no specific preventive measures have been defined. This lack of preventive measures is of particular relevance for the very unique population of perinatally-acquired HIV-infected patients, especially since these individuals have a lifelong exposure to ART and the deleterious effects of chronic inflammation associated to the infection. The aim of this study was to assess the prevalence of metabolic abnormalities, including dyslipidemia and insulin resistance, within a cohort of perinatally-acquired HIV-infected adolescents, and to determine possible associated clinical factors that could potentially be used to identify subjects at increased risk for premature cardiovascular disease.

## Methods

### Study design and eligibility criteria

We present results from a cross-sectional analysis performed in 2009, within a prospectively- followed CoRISpe-Madrid Cohort of Pediatric HIV-infected children and adolescents that started on 2002.

Inclusion criteria included: age between 12 and 20 years old, perinatally-acquired HIV infection, follow-up at one of six participating centers and at least one visit during study period with anthropometric measurements and fasting blood samples available.

The study was approved by the Ethics Committee and Clinical Research Review Board of the participating hospitals, and written informed consent was obtained from the parents or legal guardians or from the patients when they were 18 years old or over.

### Clinical assessment and anthropometric measurements

Clinical data were recorded at the time of visit and included gender, age, ethnicity, Center for Disease Control (CDC) pediatric HIV disease stage, type of treatment and Tanner stage. All patients underwent physical examination. Height and weight were measured and body mass index (BMI) was calculated. Waist and hip circumferences were measured using a plastic non-stretchable tape, at the navel during inspiration and at the maximum protrusion of the gluteal region, respectively. All anthropometric measurements were adjusted using *z* score according to the age and gender, based on referenced Spanish growth charts [14,15]. Immuno-virological details and data on previous ART history were collected from the CoRISpe-Madrid Cohort of Pediatric HIV-infected children and adolescents database [16].

### Laboratory analyses

Fasting blood samples were drawn from all participants for real-time measurements of glycemic and lipid profile, determined at the different participating hospitals using standard enzymatic methods. Determinations included insulin and glucose levels, total cholesterol, high-density lipoprotein cholesterol [HDL-c], low-density lipoprotein cholesterol [LDL-c] and triglycerides. Simultaneously, plasma HIV-1 viral load (VL) was quantified using the Cobas TaqMan HIV-1 assay (Roche Diagnostics Systems, Inc, Branchburg, NJ) with a detection limit of 50 copies/mm^3^. Absolute count and percentage of CD4 and CD8 T-cell were concomitantly measured using flow cytometry.

According to the American Diabetes Association, impaired fasting glucose was defined as glucose ≥ 100 mg/dl [17]. Insulin resistance was calculated using the homeostasis model assessment of insulin resistance (HOMA-IR = fasting insulin (microU/ml) x fasting glucose (g/dl)/405) [18]. As no pediatric reference values are available for HOMA-IR and/or fasting insulin levels, we used the cut-off points defined by García-Cuartero *et al.* [19] as the 90th percentile adjusted for sex and Tanner stage for pediatric reference population in Spain. Hypertriglyceridemia was defined by the presence of plasma triglycerides ≥ 150 mg/dl, hypercholesterolemia by total cholesterol ≥ 200 mg/dl, increased LDL-c ≥ 130 mg/dl, and low HDL-c if levels < 35 mg/dl based on current recommendations of the American Academy of Pediatrics [20].

### Statistical analyses

Continuous variables were expressed as median and interquartile range (IQR), and categorical variables as counts and percentages. Mann–Whitney *U* test were used for independent two-group comparisons in continuous variables and χ^2^ or Fisher’s exact test were used to compare categorical variables.

Adjusted analyses: All variables independently associated were included in the multivariate analysis, as well as those variables considered clinically relevant. Multivariate logistic regression model was used to study the association of ddI exposure with increased LDL-c, adjusted for potential confounders including age, sex, Tanner stage, weight PI and NRTI treatment length and CD4 nadir. Multivariate logistic regression model was used to study the association of abdominal circumference Z score with increased HOMA-IR, adjusted for potential confounders including age, sex, Tanner stage, weight, PI and NRTI treatment length and CD4 nadir. Interactions between predictor variables were evaluated but no significant results were found, and were not included in the model. All statistical analyses were performed using SPSS software 20.0 (IBM, *SPSS*, Chicago, IL, USA).

## Results

### Study population and clinical characteristics

During the study period there were 214 patients followed up in CoRISpe-Madrid Cohort of Pediatric HIV-infected children and adolescents. One hundred and four adolescents and young adults who met inclusion criteria were eligible, and five patients refused to participate in the study. Ninety-nine were enrolled, with a median age of 15.3 years [13.6-16.7] and 57.6% (n = 57) were female. Main characteristics at the time of the assessment are shown in Table 1. At the time of inclusion adolescents were either receiving HAART (n =92; 92.9%) or off therapy (7%). Twenty-eight percent of individuals had received 5 or more different ART regimens. Only one patient was receiving lipid-lowering therapy (ezetimibe) at the time of the study. Seven patients presented with hepatitis C virus co-infection.

**Table 1:** **Main characteristics of study participants**

**Variable**	**N = 99**
**Age (years)**	15.3 [IQR:13.6-16.7]
**Ethnicity**	
**Caucasian**	83 (85.6)
**African**	5 (5.1)
**Hispanic**	2 (2.1)
**Roma**	2 (2.1)
**Other**	5 (5.1)
**Female**	57(57.6)
**CDC clinical stage**	
**A**	25 (25.5)
**B**	29 (29.6)
**C**	44 (44.9)
**CDC immunological stage**	
**1**	2 (2.1)
**2**	31 (31.3)
**3**	66 (66.6)
**Tanner stage**	
**1**	4(4.1)
**2**	8 (8.1)
**3**	14 (14.1)
**4**	18 (18.1)
**5**	55(55.6)
**Weight (kg)**	51.1 [44.7-59.2]
***Weight Z score***	−0.2 [−0.79-0.76]
**Height (cm)**	160 [153.5-166.9]
***Height Z score***	−0.03 [−1-0.7]
**BMI**	19.9 [18.3-22.3]
**Age at menarche (years)**	12.0 [11.7-13.0]
**Treatment**	
**HAART**	92 (92.9)
**No treatment**	7 (7.1)
**Total ARV exposure (years)**	11.4 [9.1-12.8]
**HAART exposure (years)**	9.4 [6.7-10.7]
**PI exposure (months)**	90.4 [44.1-127.8]
**NNRTI exposure (months)**	39.8[3.3-85.7]
**Type of treatment**	
**2 NRTIs + 1 PI**	35 (38.1)
**2 NRTIs + 1 NNRTI**	28 (30.4)
**2 NRTIs + 1 PI +1 NNRTI**	21 (22.8)
**Other**	8 (8.7)
**Viral load (copies/ml)**	50 [50–200]
**Viral load (<50 copies/ml)**	66 (66.7)
**CD4 count (cs/mm** ^**3**^ **)**	737.0 [503.8-975.5]
**CD4 %**	33 [25.4-38.5]
**CD4 nadir (cs/mm** ^**3**^ **)**	252 [102–442]

Continuous variables were expressed as median and interquartile range (IQR) and categorical variables were expressed as counts (N) and percentages (%).

*Abbreviations*: *IQR* interquartile range, *CDC* centers for disease control and prevention, *HAART* highly active antiretroviral therapy, *ARV* antiretroviral, *BMI* body mass index, *NRTI* nucleoside reverse transcriptase inhibitors, *NNRTI* non-nucleoside analog, *PI* protease inhibitor.

Adherence was assessed by physician’s interview in all patients at the time of clinical visit.

### Lipid abnormalities and association with clinical variables

Twenty-five ( 25/90 27.2%) children had cholesterol levels over 200 mg/dl, 22/85 (25.9%) LDL-c over 130 mg/dl, 13/92 (14.1%) HDL-c under 35 mg/dl and 39/98 (39.8%) triglycerides over 150 mg/dl. Median values for total cholesterol, LDL-c, HDL-c cholesterol, triglycerides, glucose, insulin and HOMA are shown in Table 2.

**Table 2:** **Main metabolic results**

**Variable**	
**Total cholesterol (mg/dl)**	173.5 [IQR:151.8-202.5]
**Total cholesterol > 200 mg/dl**	25/90 (27.2)
**HDL-cholesterol (mg/dl)**	47.0 [41.3-59.8]
**HDL-c < 35 mg/dl**	13/92 (14.1)
**LDL-cholesterol (mg/dl)**	98.0 [79.3-128.3]
**LDL-c > 130 mg/dl**	22/85 (25.9)
**Triglycerides (mg/dl)**	116.0 [76.0-194.5]
**Triglycerides > 150 mg/dl**	39/98 (39.8)
**Fasting glycemia (mg/dl)**	87.0 [82–93.5]
**Fasting hyperglycemia (>100 mg/dl)**	4/87 (4.6)
**Insulin (U/ml)**	12.2 [8.3-22.8]
**Hyperinsulinemia**	26/85 (30.6)
**HOMA**	2.7 [1.8-4.7]
**Insulin resistance**	26/85 (30.6)

Continuous variables were expressed as median and interquartile range (IQR) and categorical variables were expressed as counts (N) and percentages (%).

*Abbreviations*: *IQR* interquartile range, *HDL-c* high-density lipoprotein cholesterol, *LDL-c* low-density lipoprotein cholesterol, *HOMA* homeostasis model assessment.

We first explored factors associated with the presence of increased LDL-c. In the univariate analysis, the presence of LDL-c > 130 mg/dl was associated with ddI exposure (p = 0.027) but no association with stavudine (d4T) (p = 0.4) or PI (p = 0.179) exposure was found. Children with increased LDL-c presented with higher CD4 count (852 [IQR: 714.8-1124.8] vs 625,0 [490–854], p = 0.008). In the multivariate logistic regression model, adjusting by sex, age, PI and NRTIs exposure, and CD4 count, the association between current ddI use and increased LDL-c levels did not reach statistical significance (OR = 3.44 (CI95%: 0.976-12.12), p = 0.055). Regarding HDL-c, low HDL-c levels were associated with male sex (p = 0.035) and presence of larger abdominal circumference: (78 cm [73.8-82.2] vs 72.75 cm [68.2-79.3]; p = 0.04), but no association with treatment was found.

Children with hypertriglyceridemia presented with higher CD4 count (831 [586–1115.2] vs. 660 [477–900], p = 0.026) and higher levels of triglycerides were also found in children receiving PI therapy (128.5 mg/dl [88.7-196.7] vs. 85 mg/dl [57–167.5], p = 0.022).

### Insulin and HOMA

A total of 26 (30.6%) children presented with insulin levels and HOMA-IR values above the 90^th^ centile, adjusted by sex and Tanner stage. However, only 4 (4.6%) adolescents had a fasting glucose greater than 100 mg/dl.

**Figure 1:**
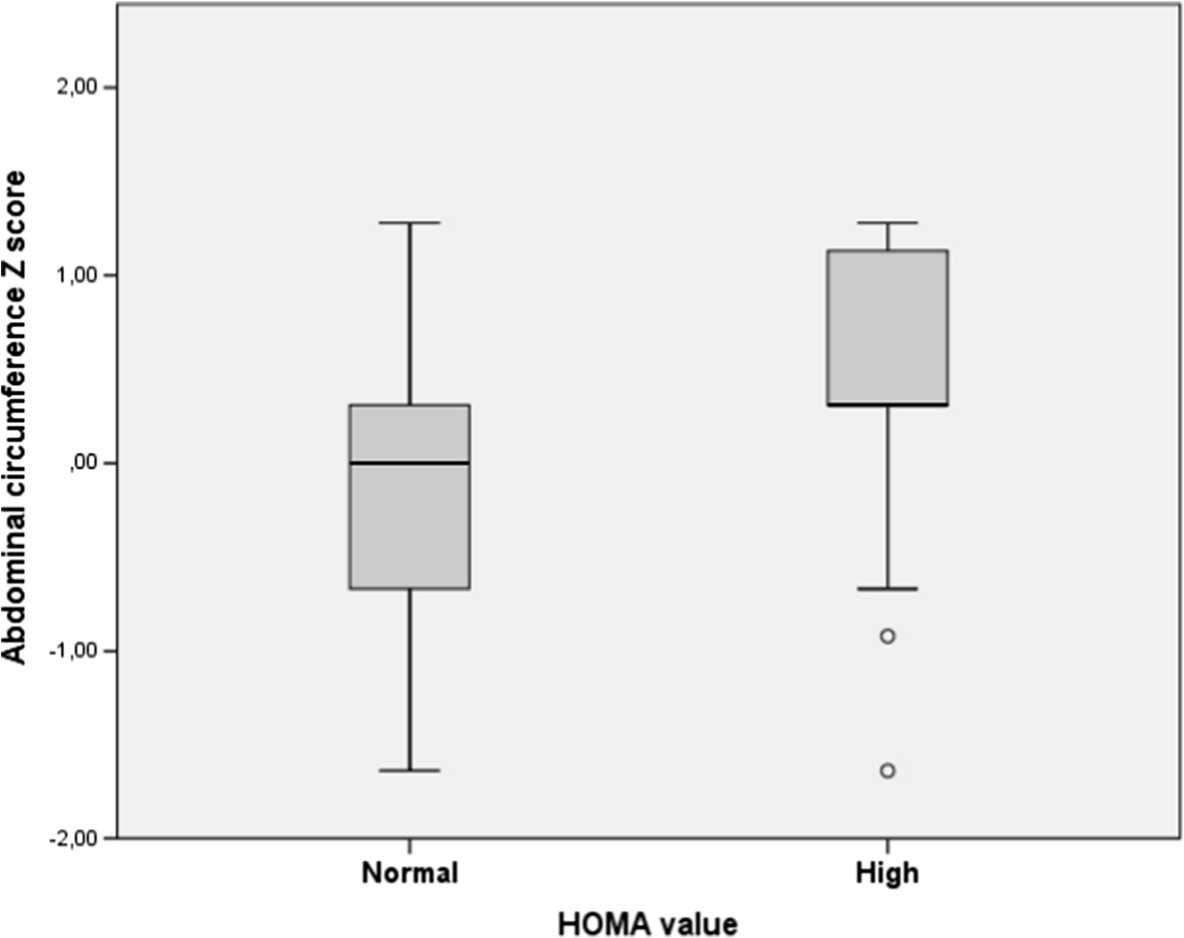
**Waist circumference** ***Z score*** **in children with and without elevated HOMA-IR values according to sex and Tanner stage.**

In the univariate analysis the presence of elevated HOMA-IR values was associated with greater waist circumference Z score (0.31 [IQR: 0.31-1.13] vs. 0 [−0.67-0.31], (p = 0.016) (Figure 1). Children with elevated HOMA-IR values had higher nadir CD4 count and percentage; 363 CD4 mm [IQR: 170–556.8] vs. 197 [88–391]; (p = 0.015) and 17.5% [10.8%-23.8%] vs. 12% [4%-16%]; (p = 0.003) respectively. No association with current or previous ART exposure, ethnicity, BMI, weight, height, waist-to-hip ratio, current CD4 count or CDC pediatric HIV disease stage was found. No association with elevated HOMA-IR was found in children with hepatitis C virus co-infection (p = 0.193).

In the multivariate logistic regression analysis, higher HOMA-IR values were associated with higher waist circumference Z score OR: 3.92(CI95%: 1.15-13.4) (p = 0.03), adjusted for sex, age, weight, Tanner stage and PI and NRTI treatment length, and CD4 nadir (Table 3).

**Table 3:** **Multivariate logistic regression model for insulin resistance and abdominal Z score adjusted for sex, age, weight, Tanner stage, CD4 nadir and PI and NRTI treatment length**

	**OR**	**CI95%**		**P value**
**Sex**	0.87	0.19	4.3	0.891
**Age**	1.72	0.97	3.01	0.056
**Weight**	0.98	0.89	1.08	0.752
**Tanner stage**	0.30	0.13	0.71	0.006
**PI treatment length**	0.99	0.98	1.0	0.258
**NNRTI treatment length**	0.99	0.97	1.005	0.158
**CD4 nadir**	1.05	0.96	1.153	0.252
**Abdominal circumference** ***Z*** **score**	3.92	1.15	13.4	0.003

*Abbreviations*: *HOMA* homeostasis model assessment, *PI* protease inhibitor, *NNRTI* non-nucleoside reverse transcriptase inhibitors.

## Discussion

In this cross-sectional study in a cohort of perinatally-acquired HIV-infected adolescents and young adults on ART, we found a high prevalence of abnormal levels of lipids in serum and insulin resistance. Up to 40% of the study participants showed hypertriglyceridemia, 27.2% increased levels of total cholesterol, 25.9% elevated LDL-c cholesterol and 14.1% of the subjects had low HDL-c. In addition, a third of the study participants had insulin and HOMA-IR levels above the 90^th^ centile. PI exposure was associated with hypertriglyceridemia, but not with increased levels of total cholesterol, LDL-c or HDL-c, and no association was found with the presence of insulin resistance. A strong correlation was seen between HOMA-IR values and Z score -adjusted waist circumference; this correlation remained significant after adjustment by potential confounders.

HIV-infected children can expect to live for many years since the introduction of HAART. However, several studies have reported a high prevalence of metabolic disorders in this unique adolescent population and there is growing concern about the long-term complications in children with lifelong exposure to ART. Dyslipidemia and insulin resistance have been widely described among ARV-treated children and adolescents, although the prevalence of these disorders differs significantly among studies [1,2,21,22]. The use of diverse ART regimens according to different national guidelines and availability may explain some of the differences found. However, socio-demographic factors, and especially factors related to diet, may also be partially responsible for this divergence in results. In the Spanish pediatric population, the reported prevalence of hypercholesterolemia(>200 mg/dl) is estimated to be 19.2-26.6% and 13-22% for elevated LDL-c (>130 mg/dl) and, in the Madrid area, 7.7% of children presented with triglycerides over 100 mg/dl [23]. We found similar rates of hypercholesterolemia (27.2%) and elevated LDL-c (14.1%), but a higher rate of hypertriglyceridemia (39,8%) in perinatally-acquired HIV-infected adolescents and young adults.

Recently, data from a cross-sectional analysis in the Spanish cohort of HIV-infected children have been published. Dapena *et al.* [2] found similar lipid abnormalities in Spain, as well as increased rate of insulin resistance (19.9%) compared to other studies. The prevalence of IR in our study (30.6%) is even higher than the overall rate in this Spanish cohort of HIV-infected children. Results from studies addressing this issue in different settings have described much lower prevalence of IR (6,5-6,8%) in HIV-infected children [4,24]. In contrast, studies including perinatally-acquired HIV-infected adolescents and young adults evidence higher rates of IR (15.2 -52%) [2,21,25,26]. The fact that the results presented here correspond to a cohort of adolescents may explain this high prevalence of IR, raising concerns as they suggest that the prevalence of these disorders among vertically HIV-infected individuals might increase as the population grows up.

In this study, IR was associated with the presence of higher waist circumference Z score values, both in the univariate and multivariate analysis. If these findings are further confirmed, a simple anthropometric measurement (such as waist circumference) could be used to detect those children with increased risk for metabolic abnormalities. We believe this finding could be extremely helpful, especially in resource-poor settings. An association between waist circumference and increased cardiovascular risk has been previously described in otherwise healthy adults [27,28]. Data on children and adolescents are, however, scarce [29,30]. In the special setting of pediatric HIV infection, associations between waist circumference and IR and other metabolic disorders have been studied, but results have been inconclusive. In the study by Geffner *et al*. higher total waist circumference (as well as other anthropometric measurements like BMI, waist-to-hip ratio and weight Z score) was associated with IR [26], but in another study including only adolescents, waist-to-hip ratio remained the only independent predictor of IR (HOMA-IR >4) in the multivariate logistic regression model [31]. Aldrovandi *et al.* found no statistically significant differences in waist circumference Z score in HIV-positive children (on PI or NNRTI treatment) compared to uninfected children and adolescents [1]. Interestingly in this study, HIV-infected children presented with higher HOMA-IR median values despite lower height, weight and BMI Z scores than healthy children [1]. Recently, the study by Hazra *et al.* found no relationship between anthropometric measurements and the presence of IR [4]. The fact that most studies used a single HOMA-IR cutoff value to define IR may explain most of the differences found among these studies. HOMA-IR reference values are not well defined in childhood and they are known to be influenced by age, gender and Tanner stage. In fact, Geffner *et al.* found that children at Tanner stage 5 were 4 times more likely to be diagnosed with IR compared to children at Tanner stages 1–3 [26]. In our study, in an attempt to more accurately identify patients at higher risk of IR, elevated HOMA-IR was defined according to the 90^th^ centile of the Spanish pediatric population, using a specific cutoff value according to gender and Tanner stage [19], and, therefore, we believe our results may be more accurate. Abdominal circumference also changes significantly from children to adolescents and young adults. Interestingly, Miller *et al.* found that HIV-infected children presented with similar total waist circumference compared to healthy controls despite having lower weight, height and BMI Z scores [3]. These findings suggest a disproportionate higher waist circumference in HIV-infected children due to a fat redistribution. We think that it is of paramount importance to use waist circumference Z score adjusted by age and sex [15] when anthropometric evaluation is performed in HIV-infected children.

In this study children with IR presented with higher CD4 nadir. Previous studies have also shown that HIV-infected children with IR are not characterized by a very low nadir CD4 (absolute and percent) [26], suggesting that the development of IR might not be related to the extent of immunodeficiency, as has been suggested for other complications associated to HIV such as cardiovascular risk [32]. On the other hand, children with hypertriglyceridemia or increased LDL-c values showed higher CD4 count at the time of the assessment, which could be explained by better adherence to ARV treatment, similar to other studies previously published [2], although this issue has not been specifically addressed in our study.

This study has the inherent limitations of a cross-sectional design, and no causal inferences can be made. The rather small sample size of the study obviously limits our ability to measure the contribution of particular ARV regimens on the development of metabolic abnormalities. Socioeconomic status of the family was not investigated in this study, and this fact should have some relation with nutrition and health habits and, therefore, with metabolic profiles. Oral glucose tolerance was not performed in this study. We use the ADA’s cutoff for impaired fasting glucose (100 mg/dl), so it should be noted that other organizations define the impaired fasting glucose cutoff at 110 mg/dl. A lower cutoff for IFG could overestimate the prevalence, but only 4 patients were over 100 mg/dl and insulin resistance definition was not influenced by IFG cutoff. Lastly, the absence of a control group of uninfected children constitutes a main limitation of this study. In order to overcome this fact, a referenced-based population has been used for comparison, and parameters have been adjusted according to age, sex and Tanner stage.

## Conclusions

Despite the unquestionable benefit of the ARV treatment for vertically-acquired HIV-infected subjects, much attention has to be paid to long-term complications of a lifelong exposure to treatment in this unique population. Very high prevalence of insulin resistance and lipid abnormalities has been found in this study as well as in other studies, and specific preventive measures for this population are still lacking. If the results presented here are further confirmed, a simple measurement of waist circumference might be a reliable marker of IR in HIV-infected adolescents, which could help to easily detect those patients with an increased risk of metabolic disorders and cardiovascular disease in the future.
